# The impact of lenalidomide exposure on response and outcomes in patients with lower-risk myelodysplastic syndromes and del(5q)

**DOI:** 10.1038/s41408-018-0126-z

**Published:** 2018-09-21

**Authors:** Mikkael A. Sekeres, Arlene S. Swern, Aristoteles Giagounidis, Alan F. List, Dominik Selleslag, Moshe Mittelman, Brigitte Schlegelberger, Gudrun Göhring, Jack Shiansong Li, Mary M. Sugrue, Pierre Fenaux

**Affiliations:** 1 Cleveland Clinic, Cleveland, OH USA; 2 Celgene Corporation, Summit, NJ USA; 3 Marien Hospital Düsseldorf, Düsseldorf, Germany; 4 Moffitt Cancer Center and Research Institute, Tampa, FL USA; 5 AZ Sint-Jan Brugge-Oostende, Bruges, Belgium; 6 Tel Aviv Sourasky Medical Center, Sackler Medical School, Tel Aviv University, Tel Aviv, Israel; 7 Institute of Human Genetics, Hannover Medical School, Hannover, Germany; 8 Formerly Celgene Corporation, Summit, NJ USA; 9 Service d’Hématologie Séniors, Hôpital Saint-Louis, Université Paris 7, Paris, France

Myelodysplastic syndromes (MDS) are clonal hematopoietic malignancies that primarily affect older adults, with consequent cytopenias, blood product transfusion needs, and truncated survival^1–3^. Undertreatment of patients with International Prognostic Scoring System (IPSS) Low- or Intermediate (Int)-1-risk MDS and deletion 5q [del(5q)] may lead to insufficient correction of anemia, iron overload, compromised quality of life, and increased morbidity^4,5^. It is recommended that patients with IPSS-defined lower-risk MDS and del(5q) initiate treatment with lenalidomide at 10 mg/day^6^. Those who develop profound neutropenia or thrombocytopenia should undergo treatment interruption followed by dose reduction to manage adverse events while continuing treatment^4,6–9^.

It is not known whether initial lenalidomide dose (at 10 or 5 mg), subsequent dose reductions, or cumulative lenalidomide dose affect long-term outcomes in patients with del(5q) MDS. In this retrospective analysis, we combined data from IPSS-defined lower-risk del(5q) MDS patients treated with lenalidomide from study start in the phase 2 MDS-003 study and the phase 3 MDS-004 study to assess the impact of cumulative lenalidomide exposure on red blood cell transfusion independence (RBC-TI) ≥ 26 weeks, cytogenetic response, overall survival, and acute myeloid leukemia (AML)-free survival.

In the phase 2, open-label MDS-003 study (NCT00065156)^7^, 148 patients received lenalidomide 10 mg on days 1–21 (*n* = 46) or days 1–28 (*n* = 102) of 28-day cycles. In the phase 3, randomized, double-blind, placebo-controlled MDS-004 study (NCT00179621)^8^, 205 patients were centrally randomized using a validated interactive voice response system 1:1:1 to lenalidomide 10 mg/day on days 1–21 of 28-day cycles (*n* = 69), or lenalidomide 5 mg/day (*n* = 69) or placebo (*n* = 67) on days 1–28 of 28-day cycles. Key inclusion criteria for both studies included IPSS Low- or Int-1-risk del(5q) MDS with or without additional cytogenetic abnormalities, and RBC transfusion-dependent anemia. Outcomes (RBC-TI ≥ 26 weeks, cytogenetic response, overall survival, and AML-free survival) were analyzed by initial lenalidomide dose group, total cumulative dose during cycles 1–3, and incidence of dose reductions. Further details on study design can be found in the Supplementary material; full methodology and key results for these studies have been reported previously^7,8^.

A total of 217 patients received an initial dose of lenalidomide 10 mg (10 mg dose group) and 69 patients received an initial dose of lenalidomide 5 mg (5 mg dose group) in the MDS-003 and MDS-004 studies. Patient baseline characteristics are shown in Supplementary Table 1; details of treatment received can be found in Supplementary Table 2.

Overall, RBC-TI ≥ 26 weeks was achieved in 148 patients (51.7%) (Supplementary Table 3); rates of RBC-TI ≥ 26 weeks were 57.1% for the 10 mg dose group vs. 34.8% for the 5 mg dose group (*p* < 0.001). Of 181 evaluable patients, 103 (56.9%) achieved cytogenetic response (major or minor responses) (Supplementary Table 3): 65.2% of patients in the 10 mg dose group vs. 30.2% in the 5 mg dose group (*p* < 0.001). Median time to onset of cytogenetic response was 148 days (range: 56–707). Of the patients who achieved a cytogenetic response, 84 of 103 (81.6%) also achieved RBC-TI ≥ 26 weeks. The odds of achieving RBC-TI ≥ 26 weeks and cytogenetic response significantly increased with each 100 mg increase in the total cumulative dose received in cycle 1 and cycles 1–3 (Table 1). A greater proportion of patients underwent dose reduction in the 10 mg dose group vs. the 5 mg dose group (72.4% vs. 53.6%; *p* < 0.004). Patients with a dose reduction had a 79% greater chance of achieving RBC-TI (relative risk [RR] = 1.79, 95% confidence interval [CI]: 1.31–2.44) and a 45% greater chance of achieving a cytogenetic response (RR = 1.45, 95% CI: 1.02–2.06) vs. patients without a dose reduction (Supplementary Table 3).

**Table 1:** Effects of each 100 mg cumulative lenalidomide dose increase during cycle 1 and cycles 1–3

	Effect of each 100 mg increase in cumulative lenalidomide dose	
	Cycle 1	Cycles 1–3
Achievement of RBC-TI ≥ 26 weeks, OR (95% CI)	3.41 (2.19–5.32)	1.59 (1.37–1.85)
Achievement of cytogenetic response, OR (95% CI)	2.61 (1.50–4.56)	1.31 (1.11–1.56)
Progression to AML or death, HR (95% CI)	0.62 (0.48–0.80)	0.85 (0.78–0.92)
Death, HR (95% CI)	0.60 (0.47–0.78)	0.83 (0.77–0.91)
Progression to AML, HR (95% CI)	0.72 (0.48–1.09)	0.98 (0.86–1.11)

*AML* acute myeloid leukemia; *CI* confidence interval; *HR* hazard ratio; *OR* odds ratio; *RBC-TI* red blood cell transfusion independence

AML-free survival for patients did not significantly differ between patients in the 10 mg dose group (median: 39.2 months, 95% CI: 32.8–45.1) and the 5 mg dose group (median: 44.3 months, 95% CI: 20.1–50.8) (log-rank *p* = 0.671). However, risk of progression to AML or death was reduced by 38% for each 100 mg increase in the total cumulative dose received in cycle 1 (*p* < 0.001; Table 1). Similarly, median overall survival did not significantly differ between patients in the 10 mg dose group (41.2 months, 95% CI: 35.3–47.2) and the 5 mg dose group (41.6 months, 95% CI: 23.7–56.4) (log-rank *p* = 0.6829). Risk of death was reduced by 40% for every 100 mg increase in the total cumulative dose received in cycle 1 (*p* < 0.001; Table 1).

Both AML-free survival and overall survival were significantly longer among patients receiving a total dose of lenalidomide in cycle 1 > 210 mg vs. patients receiving ≤ 210 mg (log-rank *p* = 0.0005 for AML-free survival, log-rank *p* = 0.0002 for overall survival) (Supplementary Fig. 1). Factors associated with AML-free survival and overall survival in univariate analysis are shown in Supplementary Table 4, and results of the multivariate Cox proportional hazards models analyzing covariates as predictors of AML-free survival and overall survival are presented in Table 2. In the final multivariate model, factors associated with improved AML-free survival included higher total cumulative lena-lidomide dose in cycles 1–3 (hazard ratio [HR] = 0.88, 95% CI: 0.80–0.97, *p* = 0.012) and dose reduction (HR = 0.44, 95% CI: 0.32–0.62, *p* < 0.0001). Higher total cumulative lenalidomide dose in cycles 1–3 (HR = 0.87, 95% CI: 0.79–0.97, *p* = 0.008) and dose reduction (HR = 0.47, 95% CI: 0.33–0.66; *p* < 0.0001) were also associated with improved overall survival.

**Table 2:** Multivariate analysis of predictive factors for AML-free survival and overall survival among lenalidomide-treated patients

Baseline characteristic	AML-free survival		Overall survival	
	HR (95% CI)	*p* Value	HR (95% CI)	*p* Value
Dose reduction (time-varying)	0.44 (0.32‒0.62)	< 0.0001	0.47 (0.33–0.66)	< 0.0001
Total dose in cycles 1–3, per LEN 100 mg increase	0.88 (0.80–0.97)	0.012	0.87 (0.79–0.97)	0.008
RBC transfusion burden, units/8 weeks	1.08 (1.03–1.13)	0.0007	1.07 (1.02–1.12)	0.010
Log platelet count, ×10^9^/l	0.68 (0.47–0.81)	0.0007	0.60 (0.46–0.79)	0.0002
FAB classification (RAEB + CMML vs. RA + RARS)	1.48 (1.02–2.13)	0.037	1.45 (1.00–2.10)	0.052
Age, per year increase	1.04 (1.02–1.05)	< 0.0001	1.04 (1.03–1.06)	< 0.0001

*AML* acute myeloid leukemia; *CI* confidence interval; *CMML* chronic myelomonocytic leukemia; *FAB* French-American-British; *HR* hazard ratio; *LEN* lenalidomide; *RA* refractory anemia; *RAEB* RA with excess blasts; *RARS* RA with ring sideroblasts; *RBC* red blood cell

This analysis is the largest to examine the relationship between lenalidomide exposure, dose reduction, response, and longer-term outcomes in patients with IPSS-defined lower-risk del(5q) MDS. Higher initial and cumulative lenalidomide dose in early treatment cycles was a significant predictor of improved achievement of RBC-TI ≥ 26 weeks and cytogenetic response, as well as improved AML-free survival and overall survival. These results suggest an induction approach of starting lenalidomide therapy at a dose of 10 mg is associated with improved outcomes in this population of patients with del(5q) MDS.

Dose reduction was also a significant positive predictor of improved AML-free survival and overall survival in multivariate analyses. The effect of dose reduction is not independent of starting dose, as patients receiving lenalidomide 10 mg were more likely to undergo dose reduction and experienced a better outcome than those starting on lenalidomide 5 mg. In a logistic regression carried out to determine whether patients who received dose reductions differed from those who did not, we found patients who started at lenalidomide 10 mg were 30% more likely to receive a dose reduction than patients who started at lenalidomide 5 mg (data not shown). Analyses of the association between dose reduction and improved survival did include those patients starting at lower doses of lenalidomide however, and retained significance in multivariable analyses. This apparent contradiction may be due to the use of lenalidomide dose reductions as part of a maintenance phase with dose reduction to 5 mg/day, particularly in response to adverse events, which may allow patients to remain on treatment for longer, leading to increased long-term drug exposure and better outcomes. It should be noted that we analyzed the effect of dose reduction, rather than dose interruption, although patients who experienced dose reduction were likely to have also had a prior dose interruption.

Limitations of this analysis include its retrospective nature and patient population heterogeneity. Patients with less favorable disease characteristics may also have received lower doses of lenalidomide initially, potentially introducing a bias in the relationship between cumulative dose and patient outcomes, an effect possibly seen in other studies^10^. However, dose reductions were in fact associated with better outcomes, suggesting results are unlikely to be affected by differences in disease severity. Another potential limitation is variation in total exposure to lenalidomide between and within treatment arms, depending on their starting dose and dosing schedule. To account for this variation, outcomes were analyzed by initial lenalidomide dose group as well as total cumulative dose during early treatment. Long-term dosing beyond cycle 3 could not be evaluated due to the potential effects of dose interruptions, dose reductions, and patient crossover on later dosing in the open-label portion of the studies.

In conclusion, it is tempting, particularly in older adults, to start at lower doses of lenalidomide to avoid treatment-related adverse events. However, our data support the use of lenalidomide at the recommended 10 mg dose as part of an initial induction phase of treatment. In the event of adverse events that cannot be managed through supportive mechanisms, this induction phase would be followed by a maintenance phase in which dose reductions are carried out per prescribing information guidelines^6^ in order to maximize exposure and treatment duration and thereby optimize outcomes in patients with lower-risk MDS and del(5q). The association between higher early cumulative lenalidomide dose, cytogenetic response, and long-term outcomes seen in this analysis suggests lena-lidomide may have a biological disease-modifying effect via targeted reduction in the del(5q) clone size.

## Electronic supplementary material

Supplementary material

Supplementary Figure 1
