# Supplementary material for: The impact of lenalidomide exposure on response and outcomes in patients with lower-risk myelodysplastic syndromes and del(5q)
Source: Blood Cancer J. 2018 Sep 21;8(10):90. doi: 10.1038/s41408-018-0126-z (PMC6173782; doi:10.1038/s41408-018-0126-z)
Supplement: Supplementary file 1 — Supplementary material [file 41408_2018_126_MOESM1_ESM.docx]

**SUPPLEMENTARY INFORMATION**

**Supplementary Methods**

*Study design*

For this analysis, patients assigned to the lenalidomide 10 mg dose group received an initial dose of lenalidomide 10 mg daily on days 1–21 or days 1–28 of 28-day cycles; patients assigned to the lenalidomide 5 mg dose group received an initial dose of lenalidomide 5 mg on days 1–28 of 28-day cycles. Subsequent changes in dose or schedule did not affect dose group assignment. MDS-004 patients in the placebo or lenalidomide 5 mg dose groups without at least a minor erythroid response by week 16 of study, or who had an erythroid relapse, could cross over to the 5 mg or 10 mg dose groups, respectively. This analysis did not include patients randomized to placebo in MDS-004 who later crossed over to receive lenalidomide. Dose reductions were permitted to manage grade ≥ 3 adverse events in the MDS-003 study or dose-limiting adverse events in the MDS-004 study, including grade 4 thrombocytopenia or neutropenia. Both the MDS-003 (NCT00065156) and MDS-004 (NCT00179621) studies conformed to the Declaration of Helsinki and were approved by individual institutional review boards of all participating institutions; all patients provided written informed consent.

*Outcome measures and statistical analyses*

Red blood cell transfusion independence (RBC-TI) ≥ 26 weeks and cytogenetic response were assessed using International Working Group (IWG) 2000 and 2006 criteria.^1,2^ Central cytogenetics review was performed at baseline, week 24, and week 48 for MDS-003, and at baseline, week 24, and every 24 weeks thereafter for MDS-004. Major cytogenetic response was defined as absence of cytogenetic abnormalities if pre-existing abnormalities were present; minor response was defined as ≥ 50% reduction in abnormal metaphases. Both definitions refer to all cytogenetic abnormalities present, including del(5q). Rates of cytogenetic response and RBC-TI ≥ 26 weeks were assessed by lenalidomide dose group, incidence of dose reductions, and each 100 mg increase in total cumulative lenalidomide dose received during cycle 1 and cycles 1–3. Acute myeloid leukemia (AML)-free survival (time from randomization to diagnosis of AML or death) and overall survival (time from randomization to death) were analyzed by lenalidomide dose group, total cumulative lenalidomide dose received in cycle 1, each 100 mg increase in total cumulative lenalidomide dose received during cycle 1 and cycles 1–3, and incidence of dose reductions. AML was defined by French-American-British (FAB) criteria. ^3^

Prognostic factors associated with AML-free survival and overall survival were identified using Cox proportional hazard models. The best multivariate model was selected by starting with all covariates associated with AML-free survival and overall survival at a *p* < 0.10 identified from the univariate model. All possible best model selection and stepwise procedures, the Akaike information criterion (AIC), and clinical judgement were used to select the final model. Specific variables considered for this analysis, including variables associated with outcomes of interest in previous studies,^4,5^ were age (years), sex, IPSS risk classification, FAB classification (refractory anemia with excess blasts [RAEB] or chronic myelomonocytic leukemia [CMML] versus refractory anemia [RA] or refractory anemia with ring sideroblasts [RARS] ), RBC transfusion burden (per unit increase), number of additional cytogenetic abnormalities [isolated del(5q), del(5q) plus 1 additional abnormality, and del(5q) plus ≥ 2 additional abnormalities], platelet count (×10^9^/l), hemoglobin level (g/dl), incidence of dose reduction, total cumulative lenalidomide dose in treatment cycle 1, and total cumulative lenalidomide dose in treatment cycles 1–3.

Data cutoff dates were Oct 1, 2010 for MDS-003 and Nov 26, 2012 for MDS-004.

The study sponsor and funder, Celgene Corporation, worked with the investigators to design this analysis and to analyze and interpret the data. This manuscript was written and approved by all the authors with editorial assistance from a professional medical writer funded by the sponsor. All authors had full access to all the data used in this analysis, and the corresponding author (MAS) had the ﬁnal responsibility for the decision to submit for publication.

**References**

1. Cheson BD *et al*. Report of an international working group to standardize response criteria for myelodysplastic syndromes. *Blood* 2000; **96**: 3671–3674.
2. Cheson BD *et al*. Clinical application and proposal for modification of the International Working Group (IWG) response criteria in myelodysplasia. *Blood* 2006; **108**: 419–425.
3. Bennett JM *et al*. Proposals for the classification of the myelodysplastic syndromes. *Br J Haematol* 1982; **51**: 189–199.
4. Fenaux P *et al*. A randomized phase 3 study of lenalidomide versus placebo in RBC transfusion-dependent patients with Low-/Intermediate-1-risk myelodysplastic syndromes with del5q. *Blood* 2011; **118**: 3765–3776.
5. List AF *et al*. Extended survival and reduced risk of AML progression in erythroid-responsive lenalidomide-treated patients with lower-risk del(5q) MDS. *Leukemia* 2014; **28**: 1033–1040.

**Figure legend**

**Supplementary Fig. 1** AML-free survival (**A**) and overall survival (**B**) by total cumulative lenalidomide dose in cycle 1

*AML* acute myeloid leukemia

**Supplementary Table 1 Baseline characteristics of lenalidomide-treated patients**

| **Patient characteristic** | **Lenalidomide-treated patients (*N* = 286)** |
| --- | --- |
| Median age, years (range) | 69.0 (36.0–95.0) |
| Female, *n* (%) | 199 (69.6) |
| IPSS risk category (central review), *n* (%) | *n* = 286 |
| Low | 89 (31.1) |
| Intermediate-1 | 121 (42.3) |
| Intermediate-2 | 15 (5.2) |
| High | 3 (1.0) |
| Missing | 58 (20.3) |
| FAB classification (central review), *n* (%) | *n* = 235 |
| RA/RARS | 180 (76.6) |
| RAEB/CMML | 55 (23.4) |
| Median time since diagnosis, years (range) | 2.7 (0.1–29.2) |
| Median RBC transfusion burden, units/8 weeks (range) | 6.0 (1.0–25.0) |
| Number of cytopenias, *n* (%) | *n* = 285 |
| 0, 1 | 166 (58.2) |
| > 1 | 119 (41.8) |
| Median bone marrow blasts, % (range) | 3.0 (0.0–49.0) |
| Median platelet count, ×10^9^/l (range) | 229.0 (15.0–1321.0) |
| Median ANC, ×10^9^/l (range) | 2.0 (0.3–20.7) |
| Median Hb level, g/dl (range) | 8.0 (3.6–11.8) |
| Cytogenetic complexity, *n* (%) | *n* = 274 |
| Isolated del(5q) | 200 (73.0) |
| del(5q) + 1 additional abnormality | 50 (18.3) |
| del(5q) + ≥ 2 additional abnormalities | 24 (8.8) |

*ANC* absolute neutrophil count, *CMML* chronic myelomonocytic leukemia, *del(5q)* deletion (5q), *FAB* French-American-British, *Hb* hemoglobin, *IPSS* International Prognostic Scoring System, *RA* refractory anemia, *RAEB* refractory anemia with excess blasts, *RARS* refractory anemia with ring sideroblasts, *RBC* red blood cell

**Supplementary Table 2 Lenalidomide exposure in early treatment and incidence of dose reduction**

|  | **Lenalidomide 10 mg (*n* = 217)** | **Lenalidomide 5 mg (*n* = 69)** | **Total (*N* = 286)** |
| --- | --- | --- | --- |
| Dose reduction, *n* (%) |  |  |  |
| Patients with dose reduction | 157 (72.4) | 37 (53.6) | 194 (67.8) |
| Patients without dose reduction | 60 (27.6) | 32 (46.4) | 92 (32.2) |
| Total cumulative dose in cycle 1, mg | 210 (165–240) | 140 (140–140) | 190 (140–210) |
| With dose reduction | 210 (160–270) | 140 (130–140) | 169 (140–210) |
| Without dose reduction | 210 (181–210) | 140 (140–140) | 169 (140–210) |
| Total dose in cycles 1–3, mg | 410 (285–623) | 360 (240–420) | 407 (270–565) |
| With dose reduction | 409 (293–560) | 298 (213–385) | 385 (270–520) |
| Without dose reduction | 585 (250–630) | 420 (328–420) | 420 (263–630) |
| Time to first dose reduction, days^*^ | 57 (36–87) | 63 (43–92) | 57 (38–91) |
| Total time on drug, days | 442 (137–1023) | 273 (121–587) | 381 (131–986) |
| With dose reduction | 510 (204–1092) | 273 (118–579) | 448 (176–1072) |
| Without dose reduction | 135 (26–766) | 254 (135–1046) | 217 (43–777) |

**p* = 0.345 for lenalidomide 10 mg vs lenalidomide 5 mg

All values are median (interquartile range)

**Supplementary Table 3 Achievement of RBC-TI ≥ 26 weeks and cytogenetic response for patients with or without dose reductions**

| Dose reduction, *n* (%) | **Patients achieving RBC-TI ≥ 26 weeks*** | | | | | |
| --- | --- | --- | --- | --- | --- | --- |
|  | **Yes** | | **No** | | **Total** | |
| Yes | 117 (60.3) | | 77 (39.7) | | 194 (100) | |
| No | 31 (33.7) | | 61 (66.3) | | 92 (100) | |
| Total | 148 | | 138 | | 286 | |
| Dose reduction, *n* (%) | **Patients achieving cytogenetic response^†^** | | | | | |
|  | **Yes** | | **No** | | **Total** | |
| Yes | | 82 (62.1) | | 50 (37.9) | | 132 (100) |
| No | | 21 (42.9) | | 28 (57.1) | | 49 (100) |
| Total | | 103 | | 78 | | 181 |

*Relative risk of achieving RBC-TI ≥ 26 weeks = 1.79 (95% CI 1.31–2.44)

^†^Relative risk of achieving cytogenetic response = 1.45 (95% CI 1.02‒2.06)

*CI* confidence interval, *RBC-TI* red blood cell transfusion independence

**Supplementary Table 4 Univariate analysis of predictive factors for AML-free survival and overall survival among lenalidomide-treated patients**

| **Baseline characteristic** | **AML-free survival** | | **Overall survival** | |
| --- | --- | --- | --- | --- |
|  | **HR (95% CI)** | ***p* value** | **HR (95% CI)** | ***p* value** |
| Total dose in cycle 1, per LEN 100 mg increase | 0.62 (0.48–0.80) | < 0.0002 | 0.60 (0.47–0.78) | < 0.0001 |
| Total dose in cycles 1–3, per LEN 100 mg increase | 0.85 (0.78–0.92) | < 0.0001 | 0.83 (0.77–0.91) | < 0.0001 |
| RBC transfusion burden, units/8 weeks | 1.12 (1.08–1.17) | < 0.0001 | 1.11 (1.07–1.16) | < 0.0001 |
| Log platelet count, ×10^9^/l | 0.53 (0.42–0.65) | < 0.0001 | 0.50 (0.40–0.62) | < 0.0001 |
| del(5q) + ≥ 2 abnormalities vs. del(5q) + 1 abnormality + isolated del(5q) | 1.49 (1.20–1.86) | 0.0003 | 1.44 (1.15–1.78) | 0.0013 |
| FAB classification (RAEB + CMML vs. RA + RARS) | 0.64 (0.45–0.91) | 0.0121 | 0.60 (0.42–0.86) | 0.0056 |
| Age, per year increase | 1.03 (1.01–1.04) | 0.0001 | 1.03 (1.02–1.05) | < 0.0001 |
| Hb, g/dl | 0.91 (0.80–1.04) | 0.1612 | 0.90 (0.79–1.03) | 0.1308 |
| IPSS (Int-1, Int-2, High vs. Low) | 0.80 (0.58–1.12) | 0.1932 | 0.83 (0.59–1.15) | 0.2607 |
| Sex, male vs. female | 0.58 (0.43–0.78) | 0.0003 | 0.58 (0.43–0.79) | 0.0004 |

*AML* acute myeloid leukemia, *CI* confidence interval, *CMML* chronic myelomonocytic leukemia, *del(5q)* deletion (5q), *FAB* French-American-British, *Hb* hemoglobin, *HR* hazard ratio, *Int* Intermediate, *IPSS* International Prognostic Scoring System, *LEN* lenalidomide, *RA* refractory anemia, *RAEB* RA with excess blasts, *RARS* RA with ring sideroblasts, *RBC* red blood cell
