# Supplementary figures and images for: The impact of lenalidomide exposure on response and outcomes in patients with lower-risk myelodysplastic syndromes and del(5q)
Source: Blood Cancer J. 2018 Sep 21;8(10):90. doi: 10.1038/s41408-018-0126-z (PMC6173782; doi:10.1038/s41408-018-0126-z)

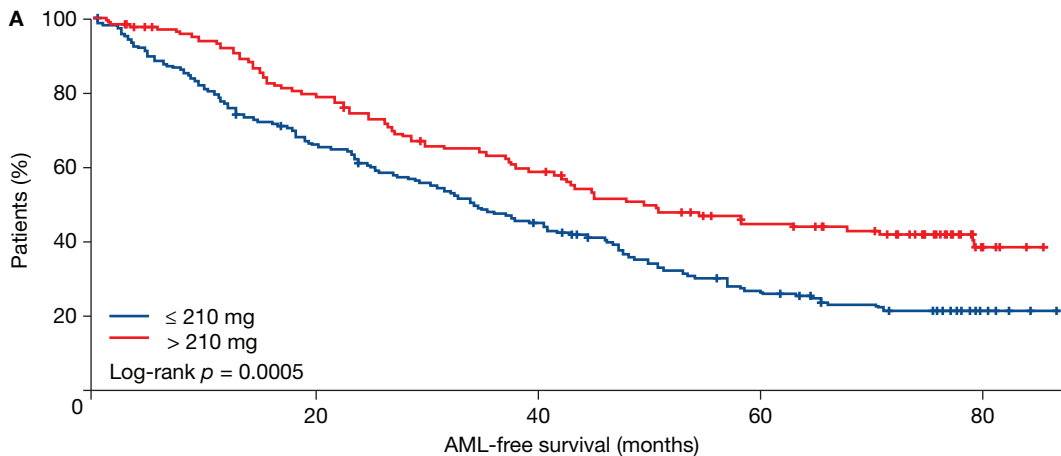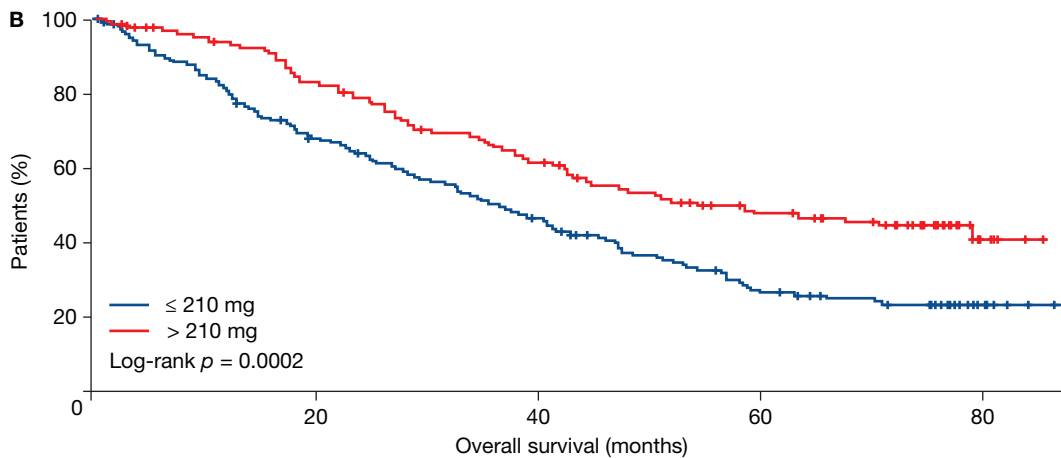

Supplement: Supplementary file 2 — Supplementary Figure 1 [file 41408_2018_126_MOESM2_ESM.pdf]
